# Case Report: Conversion therapy for initially unresectable intrahepatic cholangiocarcinoma

**DOI:** 10.3389/fonc.2026.1722347

**Published:** 2026-06-23

**Authors:** Ying-Ao Liu, Qi Huang, Xiang Gao, Wei Gao, Zeliang Hu, Fangzhou Wang, Yuanxu Qu, Yamin Zheng

**Affiliations:** 1 Department of General Surgery, Xuanwu Hospital, Capital Medical University, Beijing, China; 2 Department of General Surgery, Peking Union Medical College Hospital, Beijing, China; 3 Department of Pathology, Xuanwu Hospital, Capital Medical University, Beijing, China

**Keywords:** conversion therapy, FOLFOX, intrahepatic cholangiocarcinoma, sintilimab, surufatinib

## Abstract

**Background:**

Intrahepatic Cholangiocarcinoma (ICC) is a highly malignant tumor originating from the intrahepatic biliary epithelium, characterized by strong invasiveness and poor prognosis. For patients with unresectable advanced ICC, the current first-line standard treatment is the gemcitabine plus cisplatin regimen, but its efficacy is limited. In recent years, with the exploration of immunotherapy, targeted therapy, and various combination treatment modalities, new directions have been provided for the treatment of advanced ICC.

**Case presentation:**

This article reports a case of a patient with initially unresectable ICC who underwent conversion therapy with percutaneous portal vein embolization (PVE) combined with surufatinib (a multi-kinase inhibitor targeting VEGFR, FGFR, and CSF-1R), sintilimab (a PD-1 inhibitor), and the FOLFOX regimen (leucovorin, fluorouracil, and oxaliplatin). This approach successfully enabled radical liver resection and resulted in long-term disease-free survival. The patient was a 71-year-old male diagnosed via enhanced CT and MRI with a large liver mass accompanied by portal vein tumor thrombus. Pathological confirmation by needle biopsy confirmed intrahepatic cholangiocarcinoma. Due to extensive tumor invasion and insufficient future liver remnant (FLR), the tumor was assessed as unresectable. The treatment team adopted a multimodal conversion strategy: first performing PVE to promote compensatory hyperplasia of the FLR; subsequently administering 4 cycles of surufatinib, sintilimab combined with FOLFOX chemotherapy. Post-treatment imaging evaluation showed significant tumor shrinkage, along with an increase in FLR volume, meeting the criteria for safe radical surgery. Two weeks after stopping medication, the patient underwent right hemihepatectomy, caudate lobe resection, cholecystectomy, hepatoduodenal lymph node dissection and inferior vena cava repair. The patient recovered well postoperatively. At the last follow-up, he had achieved 28 months of disease-free survival with no evidence of local recurrence or distant metastasis.

**Conclusion:**

For initially unresectable ICC, conversion therapy with PVE combined with surufatinib, sintilimab, and the FOLFOX regimen can significantly increase the tumor resection rate and achieve long-term survival. This strategy combines high anti-tumor efficacy with clinical feasibility, providing a new approach for the conversion therapy of advanced ICC. Further larger sample studies are needed in the future to verify its efficacy and safety.

## 1 Introduction

Cholangiocarcinoma is a group of highly heterogeneous malignant epithelial tumors originating from the biliary epithelium, classified into intrahepatic cholangiocarcinoma (ICC), perihilar cholangiocarcinoma (PCC), and distal cholangiocarcinoma (DCC) (1–3). Over the past four decades, the global incidence and mortality of ICC have shown a continuous upward trend (4–6). Currently, radical surgical resection is the most effective treatment for early-stage ICC; however, most patients are diagnosed at an advanced stage, losing the opportunity for surgery (7). The median survival period for patients with advanced cholangiocarcinoma is usually less than two years, with a five-year survival rate of only about 9% (8, 9). The gemcitabine plus cisplatin (GC regimen) is the standard first-line chemotherapy regimen, but the prognosis remains poor, with a median overall survival of approximately 11 months, thus urgently necessitating the exploration of new treatment strategies (10, 11).

In recent years, with advances in precision medicine, immunotherapy, by reducing the immune escape ability of tumor cells or enhancing the body’s anti-tumor immune response, has shown significant potential in the treatment of malignant tumors (12, 13). Immune checkpoint inhibitors (ICI) have achieved success in various malignancies, including hepatocellular carcinoma (HCC), promoting their widespread research application in biliary tract cancers (BTC) (14–17). Although ICI monotherapy provides survival benefits for some patients, its overall efficacy remains limited. Currently, numerous clinical studies are dedicated to exploring strategies combining ICI with chemotherapy, anti-angiogenic drugs, or local therapies, with preliminary results showing encouraging anti-tumor activity and tolerable safety (18).

Surufatinib is a novel oral tyrosine kinase inhibitor with a unique dual mechanism of anti-angiogenesis and immune regulation (19, 20). It inhibits the formation of tumor neovasculature by targeting vascular endothelial growth factor receptor (VEGFR) and fibroblast growth factor receptor (FGFR); simultaneously, it can modulate tumor-associated macrophages by inhibiting colony-stimulating factor-1 receptor (CSF-1R), improving the immune microenvironment, and promoting immune activation. Several recent retrospective studies have shown that the combination of anti-PD-1 antibodies and surufatinib demonstrates significant efficacy in advanced cholangiocarcinoma (21–24).

This article reports a case of a patient with unresectable advanced ICC who, after receiving treatment with portal vein embolization (PVE), surufatinib combined with sintilimab (PD-1 inhibitor), and FOLFOX (leucovorin, fluorouracil, and oxaliplatin) chemotherapy, successfully achieved conversion and underwent radical surgical resection, obtaining long-term disease-free survival.

## 2 Case presentation

A 71-year-old male patient was admitted to our hospital in February 2023, with the chief complaint of “discovering liver space-occupying lesions on physical examination more than 4 months ago.” In October 2022, an abdominal ultrasound suggested liver space-occupying lesions. In December 2022, an enhanced CT of the upper abdomen indicated low-density lesions in the liver, possibly malignant (**Figure 1**); further diagnosis and treatment were delayed due to the COVID-19 pandemic. In February 2023, an MRI at our outpatient department showed liver space-occupying lesions (approximately 9.0×5.1×8.0 cm), with unclear display of the right portal vein branch (**Figure 1**). The course of the disease was without accompanying symptoms such as abdominal pain, distension, nausea, vomiting, liver discomfort, fever, or weight loss. Past medical history included type 2 diabetes mellitus and hypertension, with no history of chronic hepatitis.

**Figure 1:**
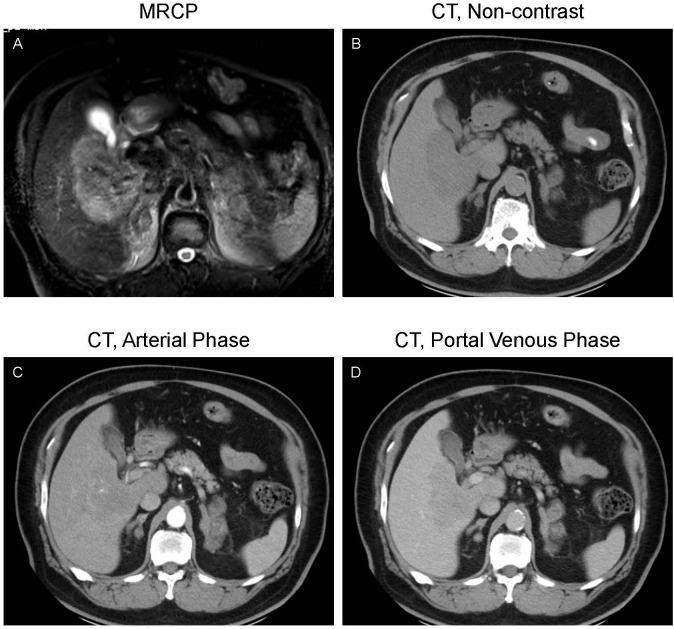
Initial imaging for diagnosis. **(A)** Magnetic resonance imaging (MRI) reveals a large space-occupying lesion in the liver (approximately 9.0 × 5.1 × 8.0 cm). The right portal vein branch is not clearly displayed, indicating possible tumor thrombus. **(B–D)** Contrast-enhanced CT scan shows low-density lesions in the liver, suggestive of a malignant tumor.

Laboratory tests after admission: complete blood count, liver and kidney function tests, and tumor markers level tests showed no significant abnormalities. Notably, the baseline serum tumor markers were within normal limits: Carcinoembryonic Antigen (CEA) was 0.95 ng/mL (reference range: 0.01–5 ng/mL), Carbohydrate Antigen 19-9 (CA19-9) was 13.15 U/mL (reference range: 0.01–37 U/mL), and Alpha-fetoprotein (AFP) was 2.23 ng/mL (reference range: 0.01–7 ng/mL). Indocyanine green 15-minute retention rate (ICG-R15) was 12.6%, Child-Pugh classification was Grade A. After obtaining informed consent, an ultrasound-guided liver biopsy was performed. Histopathological examination suggested adenocarcinoma infiltration. Immunohistochemistry results: CK8 (+), CK18 (+), CK7 (+), CK19 (+), Hepatocyte (-), ARG1 (-), CD10 (-), GPC-3 (-), HSP70 (-), GS (-), Ki-67 (approximately 8%+), CDX-2 (-), SATB2 (individual +). Mismatch repair proteins (MSH2, MSH6, MLH1, PMS2, and EPCAM) were all intact. The findings were consistent with a diagnosis of ICC.

Enhanced CT of the upper abdomen after admission showed: Irregular low-density shadows in liver segments I, V, and VI, with clear boundaries, showing progressive enhancement after contrast, less than the surrounding liver parenchyma; the right posterior branch of the portal vein was unclear. Three-dimensional reconstruction assessed the left liver volume as 579 mL (29.83% of total liver volume) (**Figure 2**); the tumor volume had increased from 124 mL two months prior to 172 mL. After multidisciplinary team (MDT) discussion, considering the large tumor volume, the presence of a single large intrahepatic lesion invading the right portal vein (tumor thrombus), and insufficient future liver remnant (FLR), radical surgery was deemed unfeasible. For this unresectable advanced ICC, the traditional GC regimen has limited efficacy; conversion therapy was planned to shrink the tumor, increase the FLR, and strive for a surgical opportunity.

**Figure 2:**
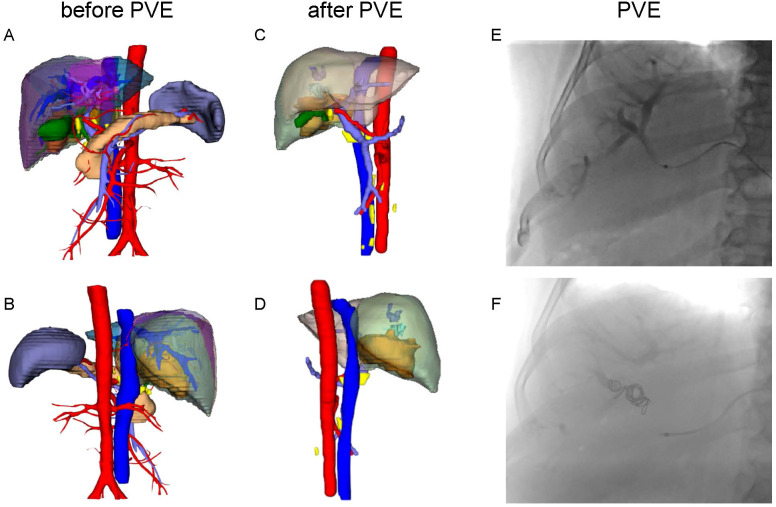
Assessment of future liver remnant (FLR) volume and response to portal vein embolization (PVE). **(A, B)** Three-dimensional volume reconstruction based on CT imaging before PVE calculated the left liver volume to be 579 mL, accounting for 29.83% of the total liver volume. **(C, D)** Follow-up enhanced CT three weeks after PVE showed an increase in the left liver volume to 847 mL (47% of total liver volume). **(E, F)** X-ray films during PVE surgery.

Treatment.

To increase the FLR, the patient underwent percutaneous portal vein embolization (PVE) on February, 2023. A follow-up enhanced CT three weeks post-PVE showed the left liver volume increased to 847 mL (47% of total liver volume), but the tumor volume also increased to 180 mL, with newly observed invasion of the hepatic hilum and inferior vena cava (**Figure 2**). ICG-R15 increased to 28.6%. The assessment indicated poor short-term results after PVE, and the patient still did not meet the conditions for surgery.

To further guide systemic treatment, genetic testing was performed on the biopsy tissue, revealing IDH1 and IDH2 mutations. Considering that the corresponding targeted drugs ivosidenib and enasidenib were not approved in China for cholangiocarcinoma indications, surufatinib, a multi-target inhibitor with anti-angiogenic and immunomodulatory effects, was selected. Due to high tumor mutational burden (TMB), it was combined with the PD-1 inhibitor sintilimab. For chemotherapy, the GC regimen was not chosen; instead, the FOLFOX regimen was administered via hepatic arterial infusion chemotherapy (HAIC) or transarterial chemoembolization (TACE) for local delivery. The overall treatment strategy was combination chemotherapy with immunotherapy and targeted therapy, with each cycle lasting 3 weeks. Imaging evaluation for efficacy and surgical feasibility was performed every 2 cycles.

The patient completed a total of 4 cycles of immunotherapy combined with targeted therapy. During this period, he underwent HAIC and TACE. Follow-up enhanced CT of the upper abdomen showed the tumor volume decreased from 172 mL to 164 mL, and the left liver volume increased to 854 mL (53% of total liver volume). ICG-R15 decreased to 20.9%. During the conversion therapy period, the patient’s tumor markers remained stable and within normal ranges (e.g., pre-operative CEA: 0.97 ng/mL, CA19-9: 16.50 U/mL). Given the non-secretory nature of this tumor, imaging responses and FLR volumetric changes were utilized as the primary measures of efficacy. Two weeks after stopping medication, the assessment confirmed eligibility for surgery.

Operation.

Surgery was performed on June, 2023. Intraoperative findings showed an enlarged liver, a palpable hard mass approximately 8×6×4 cm in the right posterior lobe with unclear boundaries, invading the left hepatic duct, artery, and portal vein. Consequently, right hemihepatectomy, left caudate lobe resection, cholecystectomy, hepatoduodenal lymph node dissection and inferior vena cava repair were performed. Postoperative histopathological examination confirmed a moderately differentiated intrahepatic cholangiocarcinoma (small duct type). The tumor tissue exhibited extensive treatment-induced changes, including large areas of necrosis, fibrous tissue proliferation, and multinucleated giant cell infiltration. The surgical margins were negative (R0 resection). Crucially, the hepatoduodenal lymph node dissection yielded 2 lymph nodes, both of which were negative for metastasis (0/2). While the tumor involved the liver capsule and demonstrated focal perineural invasion, no microvascular invasion was noted. Background liver evaluation revealed hepatocellular steatosis (**Figure 3**). Postoperative ultrasound indicated patent blood flow in the liver area, and liver and kidney functions were essentially normal. Postoperative abdominal drainage volume gradually decreased from 760 mL per day, dropping below 100 mL per day after 4 weeks. After drain removal, the patient recovered well and was discharged smoothly.

**Figure 3:**
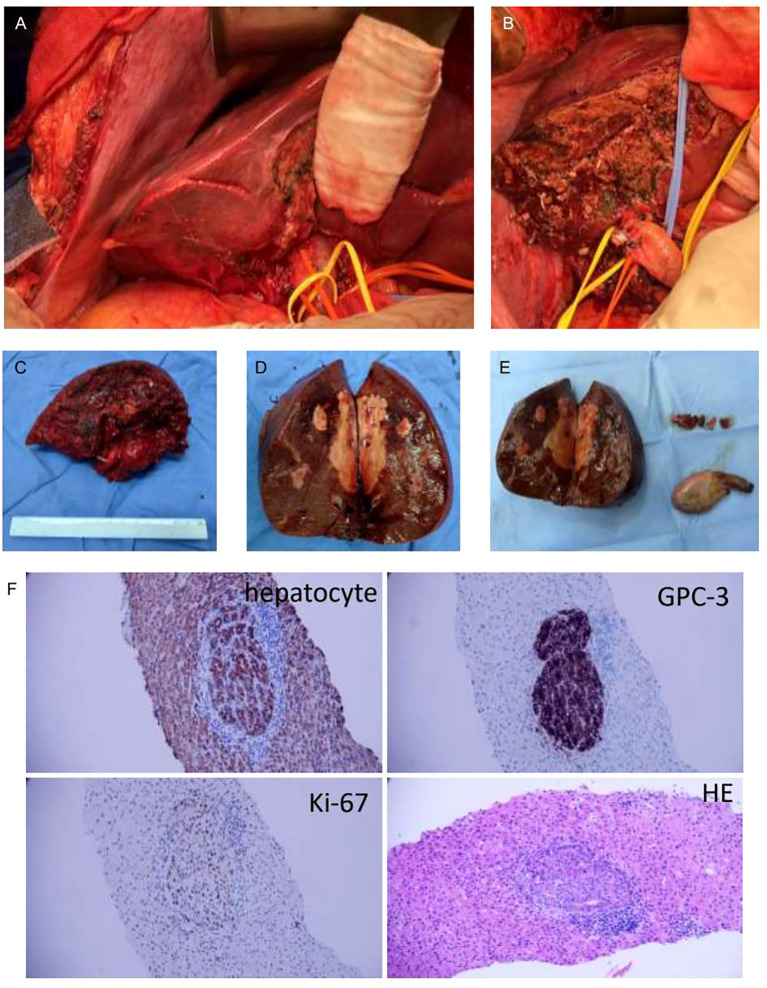
Intraoperative and postoperative pathological images of the patient. **(A, B)** Intraoperative views of the patient’s liver. **(C–E)** Gross specimens of the resected liver and gall bladder. **(F)** Photomicrograph of a representative area from the postoperative paraffin-embedded tissue section stained with hematoxylin and eosin (H&E), hepatocyte, GPC-3 and Ki-67.

Follow-up.

After surgery, the patient received 6 cycles of adjuvant therapy with surufatinib. Follow-up examinations, including upper abdominal CT or MR and tumor markers test, were performed every 3 months. Throughout the follow-up period, the tumor markers consistently remained at normal low levels (e.g., CEA: 0.74-1.16 ng/mL, CA19-9: 17.50-25.60 U/mL). As of the last follow-up (28 months post-operation), there was no evidence of local recurrence or distant metastasis. A PET-CT scan on March, 2025 (21 months post-operation) showed no metabolically hyperactive lesions. The patient has now achieved 28 months of disease-free survival. **Figure 4** shows the patient’s treatment flowchart.

**Figure 4:**
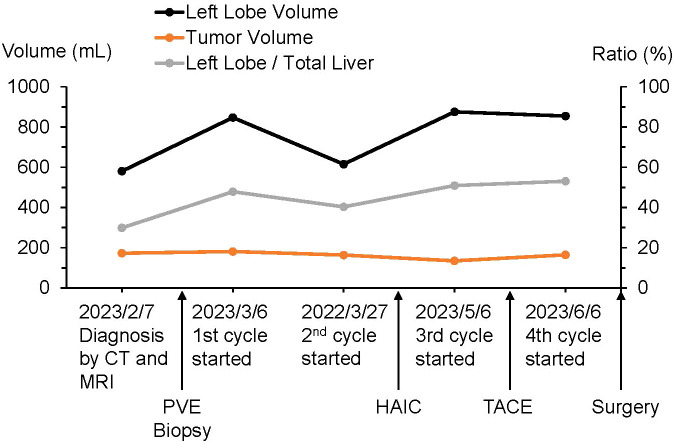
Timeline of the patient’s treatment course. This flowchart summarizes the key diagnostic and therapeutic events for the patient. Key time points include: initial diagnosis, admission and PVE, 4 cycles of conversion therapy and radical surgery.

## 3 Discussion

Intrahepatic cholangiocarcinoma (ICC) is a highly malignant biliary tract tumor with a poor prognosis, and a 5-year survival rate of only about 9% (8, 25). Early diagnosis and radical surgical resection remain the primary means for patients to achieve long-term survival. However, the early diagnosis rate of ICC is low, with only about 20-30% of patients meeting the criteria for surgery at diagnosis; the median survival time for this group after surgery can reach 53 months (7, 26). For the approximately 70%-80% of patients with locally advanced unresectable or metastatic disease, gemcitabine combined with cisplatin (GC regimen) is the standard first-line chemotherapy regimen. Unfortunately, due to frequent chemotherapy resistance, the prognosis remains poor, with survival often limited to around one year (27, 28). Therefore, exploring more effective treatment strategies for advanced ICC has always been a focus and challenge in clinical research.

In recent years, immunotherapy has shown promise in various tumors. In the field of ICC, previous studies have shown that about 10%-20% of patients respond to immunotherapy (29). A phase III, randomized, double-blind, placebo-controlled study (TOPAZ-1) compared GC combined with durvalumab versus GC combined with placebo in the treatment of advanced biliary tract cancer (including ICC). The results showed a significant extension in median overall survival (mOS) in the durvalumab group, and subgroup analysis indicated that durvalumab demonstrated a survival benefit regardless of the patient’s region.

With the advancement of large-scale tumor genomic sequencing, the molecular mechanisms of ICC development are gradually being elucidated, leading to the discovery of a series of key driver gene mutations and corresponding targeted drugs (18, 30, 31). FGFR2 and IDH1 have become promising therapeutic targets in ICC. In a multinational, open-label, single-arm phase II study, 103 advanced ICC patients with FGFR2 fusions/rearrangements received futibatinib treatment, achieving an objective response rate (ORR) of 41.7% (43 patients), a median progression-free survival (mPFS) of 9.0 months, and a mOS of 21.7 months (32). Another multicenter, randomized, double-blind, placebo-controlled phase III study (ClarIDHy) evaluated the efficacy of the IDH1 inhibitor ivosidenib in treating IDH1-mutated cholangiocarcinoma (33). The study included 185 patients, and the results showed that the ivosidenib group significantly prolonged mPFS compared to the placebo group, with good tolerability. In a subgroup analysis focused on advanced ICC, the mOS of the ivosidenib group was also superior to the placebo group (10.3 months vs. 7.5 months), further confirming the clinical value of targeting IDH1 mutations in the corresponding patient population (34). Furthermore, surufatinib, as a novel multi-target tyrosine kinase inhibitor, inhibits tumor angiogenesis by targeting VEGFR and FGFR, while simultaneously modulating tumor-associated macrophages by inhibiting CSF-1R, thereby improving the immune microenvironment (19). This drug has shown significant efficacy in the treatment of pancreatic neuroendocrine tumors, and the combination of surufatinib with PD-1 antibodies has demonstrated synergistic effects in various solid tumors (20, 35–38). Multiple studies are currently exploring its efficacy in cholangiocarcinoma. A single-arm, multicenter, open-label phase II study conducted in China evaluated the effect of surufatinib monotherapy in 39 cholangiocarcinoma patients. The 16-week progression-free survival rate was 46.33%, indicating moderate clinical activity and favorable tolerability and safety (23).

Despite significant progress in both immunotherapy and targeted therapy, the efficacy of monotherapy remains suboptimal. Increasing evidence supports that combination therapy can yield better outcomes. A single-center, single-arm phase II study reported results of toripalimab, lenvatinib, combined with the GEMOX (gemcitabine + oxaliplatin) regimen in treating advanced ICC: among 30 evaluable patients, the ORR reached 80% (23 partial responses, 1 complete response), with mOS and mPFS reaching 22.5 months and 10.2 months, respectively (39). Another randomized, double-blind, placebo-controlled phase III trial (KEYNOTE-966) compared pembrolizumab combined with GC versus GC combined with placebo in 1069 patients with advanced biliary tract cancer (including ICC). The results showed a significant extension in mOS in the pembrolizumab group (12.7 months vs. 10.9 months) (40). These studies suggest that combination therapy strategies are promising superior options for advanced ICC.

Based on this research background, our hospital treated the advanced ICC patient reported herein. Given the lack of a clear, highly effective, and unified treatment plan for advanced ICC, we developed an individualized treatment plan through MDT discussion, considering the patient’s specific situation. Acknowledging the limited survival with drug therapy alone, our goal was to control tumor growth, reduce tumor volume, and increase the future liver remnant (FLR) through preoperative conversion therapy, aiming to ultimately achieve an opportunity for radical surgery. To accomplish this goal, we decided on a comprehensive strategy combining systemic drug therapy with local interventional therapy.

For systemic drug therapy, we chose a triple combination of chemotherapy plus immunotherapy and targeted therapy. Although traditional chemotherapy has limited efficacy in advanced ICC, we believe this stems more from the development of resistance rather than complete drug ineffectiveness. In fact, chemotherapy is the cornerstone of systemic therapy. Immunotherapy and targeted therapy, by modifying the tumor microenvironment, may enhance chemotherapy sensitivity and delay resistance. In terms of administration route, to increase local drug concentration in the tumor and reduce systemic toxicity, we prioritized Hepatic Arterial Infusion Chemotherapy (HAIC) and Transarterial Chemoembolization (TACE) over systemic intravenous chemotherapy. Although GC is the standard first-line regimen, it requires intravenous administration and is unsuitable for local chemotherapy. Therefore, we selected the second-line FOLFOX regimen (leucovorin, fluorouracil, and oxaliplatin). In fact, FOLFOX also demonstrates good tumor-killing effects and tolerable side effects. After receiving two cycles of immunotherapy (sintilimab) + targeted therapy (surufatinib) combined with one session of HAIC local chemotherapy, the patient’s tumor volume significantly shrank, validating the effectiveness of this strategy. However, after the third cycle (continuing immunotherapy + targeted therapy) and receiving TACE (initially intended to further enhance the effect of PVE and increase FLR), the tumor volume rebounded. We analyzed that prolonged treatment time might lead to drug resistance; additionally, hepatic artery embolization post-TACE might hinder subsequent systemic drugs from reaching the tumor area, affecting the overall efficacy of the combination therapy. The choice of targeted drug was based on genetic testing results, which showed IDH1 and IDH2 mutations. Although targeted drugs for IDH1 mutation (such as ivosidenib) have shown benefit in studies, they are not yet approved in China for the ICC indication. Therefore, we selected surufatinib, which has multi-target inhibitory effects, aiming to inhibit tumor angiogenesis and synergistically regulate the immune microenvironment.

Insufficient FLR at admission was another key factor preventing surgery. While an FLR of 29.83% might be marginally acceptable for a healthy liver, a minimum FLR of 30%-40% is strictly required for patients with advanced age, diabetes, or underlying hepatic steatosis (as later confirmed by pathology in our patient) to avoid post-hepatectomy liver failure. Furthermore, the presence of the large tumor burden and major vascular invasion strongly predicted early recurrence, classifying the tumor as borderline resectable or initially unresectable. Besides drug therapy to control the tumor, the main methods to increase FLR include Associating Liver Partition and Portal vein ligation for Staged hepatectomy (ALPPS) and Percutaneous Portal Vein Embolization (PVE). ALPPS acts rapidly (about 3–5 days) but carries higher surgical risk; PVE is relatively safer, but FLR hyperplasia is slower (taking weeks), during which there is a risk of tumor progression, although combined drug therapy can somewhat control this risk. After comprehensively assessing the patient’s liver function and overall condition, we chose the safer PVE. However, during the 3-week hypertrophy window post-PVE, the tumor exhibited highly aggressive biological behavior, with its volume increasing and new vascular invasion emerging. This rapid progression definitively proved that local intervention alone was insufficient, and intensive systemic conversion therapy was absolutely necessary to halt progression and achieve a curative R0 resection. Reviewing this case, we believe that combining PVE with TACE requires caution: first, simultaneous embolization of the portal vein and hepatic artery may excessively impair liver blood supply, affecting liver regeneration and function; second, hepatic artery embolization impedes subsequent systemic drugs (especially targeted and immunotherapeutic agents) from reaching the tumor area, weakening the treatment effect.

It is noteworthy that during conversion therapy, the timing of surgery needs to be actively and meticulously assessed. This case suggests that multi-dimensional evaluation (imaging, liver function, FLR, etc.) should be performed promptly after each treatment cycle. This patient reached the optimal surgical window after two cycles of drug therapy: the tumor had significantly shrunk, and the FLR already exceeded 50%. Conversely, the tumor regrew during continued drug therapy, possibly related to acquired resistance. Determining the precise timing for surgery requires accumulating more clinical experience and conducting further research for clarification.

In summary, we reported a case of a patient with initially unresectable advanced ICC who successfully achieved conversion through PVE to increase FLR, combined with surufatinib, sintilimab, and the FOLFOX regimen (via HAIC/TACE) to control the tumor, underwent radical surgery, and ultimately achieved long-term disease-free survival. The results of this case indicate that this comprehensive strategy integrating local intervention (increasing FLR) with systemic combination drug therapy (controlling the tumor) is feasible and offers a potential treatment option for patients with advanced ICC. Its definitive safety and efficacy require further validation through larger-scale studies.

## Data Availability

The original contributions presented in the study are included in the article/supplementary material. Further inquiries can be directed to the corresponding author.
